# Supplementary material for: Swine Practitioner Practices on Oral Fluid Sampling in U.S. Swine Farms: A Nationwide Survey
Source: Pathogens. 2025 Sep 16;14(9):940. doi: 10.3390/pathogens14090940 (PMC12473141; doi:10.3390/pathogens14090940)
Supplement: Supplementary file 1 [file pathogens-14-00940-s001.zip › S1_Questionnaire_pilot survey.pdf]

Characterization of current oral fluid usage in pig production systems in the United States

Elija en qué idioma desea continuar en el cuadro superior derecho
Please choose which language you would like to proceed with from the top right box

Characterization of current oral fluid in pig production systems in the United States.

Thanks for participating in this survey.

The results from this survey will be aggregated and shared in a summary report that WILL NOT include your name or company name.

You will be compensated for the time invested in completing this survey with a $25 gift card that will be sent to you.

Demographics

1. First and Last Name ____

2. Email Address ____

3. Choose the one that best describes your job position:

- ○ I am a Producer
- ○ I am a Veterinarian
- ○ I am a contract grower and provide pig care (e.g. feed, water, ventilation, treatments)
- ○ I am responsible for pig care (e.g. feed, water, ventilation, treatments)
- ○ I own the pigs and provide pig (e.g. feed, water, ventilation, treatments)
- ○ I supervise the team of individuals who are responsible for pig care (e.g., feed, water, ventilation, treatments)

4. Which company do you work for?___________________________________

5. What is your job title? __________________________

Information related to the pig barns and pigs you oversee

6. How many sites and pigs are you responsible for? Please write the total number of sites and pigs below.

- ○ SITES ____
- ○ PIGS ____

7. For the number of sites you mentioned in the previous question, please provide information regarding the number of barns per site, the number of pigs per barn and the farm type (production type) below.

Site	Number of Pig Spaces per Barn?	Farm type (e.g., Nursery, Finisher, Wean-to-Market)	Number of Barns at the site?
1			
2			
3			
4			
5			
6			
7			
8			
9			
10			
11			
12			
13			
14			
15			
16			
17			
18			
19			
20			

8. Based on the previous table, please complete the following information regarding the number of pens per barn and the number of pigs per pen.

Site	Number of Pens per Barn?	Number of Pigs per Pen?
1		
2		
3		
4		
5		
6		
7		
8		
9		
10		
11		
12		
13		
14		
15		
16		
17		
18		
19		
20		

Oral Fluids Usage Information

9. Are you familiar with oral fluid collection and its use for diagnostics?

- ◯ YES
- ◯ NO

10. Usually how often do you collect oral fluids per site?

- ◯ Daily
- ◯ Weekly
- ◯ Monthly
- ◯ Other - Please explain below:

____

11. If there are clinical signs, how often would you collect oral fluids?

- ◯ Daily
- ◯ Weekly
- ◯ Monthly
- ◯ Other - Please explain below:

____

12. Is the frequency of collecting oral fluids timed on other factors such as a new group of pigs, regional disease pressure, pigs are close to being shipped to another site/market, or another factor? Please explain:____

____

____

13. What is your primary use of oral fluids as a diagnostic sample?

- ◯ Testing for routine surveillance (regardless of clinical signs)
- ◯ Testing based on the presence of clinical signs (e.g., cough, sneeze, diarrhea, mortality)
- ◯ Testing based on other factors. Please explain:

____

14. When you collect oral fluids, how many ropes do you hang per barn?

____

15. What best describes the method you use to determine the number of ropes to hang?

- ◯ Number dictated by my production manager
- ◯ Number dictated by my veterinarian
- ◯ I decide how many ropes I need to hang
- ◯ Other. Please explain: ____

16. What is the average number of ropes used per pen?

- ◯ One per pen
- ◯ One per 2 pens (hung on the gating separating 2 pens)
- ◯ Two per pen
- ◯ Other. Please explain: ______________________________

17. What is the average number of pens sampled per barn?

____

18. What would be the average number of pigs represented by a single oral fluid-rope sample?

____

Sampling and Sample Management Procedures

19. Who usually collects the oral fluid sample?

- ◯ I do
- ◯ My Supervisor
- ◯ My Veterinarian
- ◯ Other. Please explain: ______________________________

20. What is the length of time pigs have access to the rope? Enter total of minutes below:

____

21. Do you know what the source (the store, vendor the rope originated from) and the diameter of the rope?

- ☐ Source: ______________________________
- ☐ Diameter (please include units):

____

22. Do you have a written protocol for handling/processing the ropes to collect oral fluid samples at the farm?

- ○ YES
- ○ NO

23. Once samples are collected, are they immediately:

- ○ Stored in refrigeration at the farm
- ○ Stored in refrigeration in the truck
- ○ Left in a truck until arrived at the office for refrigeration
- ○ Other. Please explain: ____________________

24. How much time (average, minimum, maximum) goes by between you collecting the oral fluids and submitting the oral fluids to the diagnostic laboratory?

- ☐ Average (Hours) ____________________
- ☐ Minimum (Hours) ____________________
- ☐ Maximum (Hours) ____________________
- ☐ I don't know

Sample Submission

25. Once samples are collected, where do they go next?

- ○ My veterinarian's clinic
- ○ My employer/pig production company's laboratory
- ○ Directly to the veterinary diagnostic laboratory
- ○ Other. Please explain: ____________________

26. When oral fluid samples are submitted, which of the following information do you include? (Check all that apply)

- ▢ Clinical signs observed
- ▢ Number of dead pigs
- ▢ Total number of pigs in the site
- ▢ Pig age
- ▢ Number of pigs with clinical signs
- ▢ The date on which clinical signs were first observed
- ▢ The Premises Identification Number **(PIN)**

Skip To: End of Block If Condition: Selected Count Is Less Than or Equal to 6. Skip To: End of Block.

End of Block: Default Question Block
