# Supplementary material for: Swine Practitioner Practices on Oral Fluid Sampling in U.S. Swine Farms: A Nationwide Survey
Source: Pathogens. 2025 Sep 16;14(9):940. doi: 10.3390/pathogens14090940 (PMC12473141; doi:10.3390/pathogens14090940)
Supplement: Supplementary file 1 [file pathogens-14-00940-s001.zip › S2_Questionnaire_final.pdf]

Characterization of current oral fluid usage in pig production systems in the US

Characterization of current oral fluid usage in pig production systems in the US.

Thanks for participating in this survey.

The results from this survey will be aggregated and shared in a summary report that WILL NOT include your name or company/clinic name.

You will be compensated for the time invested in completing this survey with a $25 gift card that will be sent to you.

Demographics

1. First and Last Name ____

2. Email Address ____

3. Choose the one that best describes your job position:

   ○ I am a Producer

   ○ I am a Veterinarian

   ○ I am a contract grower and provide pig care (e.g. feed, water, ventilation, treatments)

   ○ I am responsible for pig care (e.g. feed, water, ventilation, treatments)

   ○ I own the pigs and provide pig (e.g. feed, water, ventilation, treatments)

   ○ I supervise the team of individuals who are responsible for pig care (e.g., feed, water, ventilation, treatments)

4. Which company/clinic do you represent? __________________________________

5. What is your job title? __________________________

Information related to the pigs you oversee

6. Approximately how many pigs are you responsible for? Please write the estimated total number of sows and growing pigs.

- ○ Sows ____

- ○ Growing pigs ____

Oral Fluids (OF) Usage Information

7. Do you use oral fluids for diagnostics?

- ○ NO

- ○ YES

Skip To: End of Survey If Oral Fluids (OF) Usage Information 7. Do you use oral fluids for diagnostics? = NO

8. On average, how often do you or your team collect oral fluids per site? Please explain below your frequency of OF collection.

	GDU	Sow Farm	Growing Pig Farm
Daily	☐	☐	☐
Weekly	☐	☐	☐
Monthly	☐	☐	☐
Other - Please explain below:			

9. If there are NO clinical signs, how often would you collect oral fluids?

- ◯ Daily
- ◯ Weekly
- ◯ Monthly
- ◯ Other - Please explain below:

____

10. If there are clinical signs, how often would you collect oral fluids?

- ◯ Daily
- ◯ Weekly
- ◯ Monthly
- ◯ Other - Please explain below:

____

11. Is the frequency of collecting oral fluids timed on other factors such as a new group of pigs, replacement animals, regional disease pressure, pigs are close to being shipped to another site/market, or another factor? Please explain:

____

12. What is your primary use of oral fluids as a diagnostic sample?

- ◯ Testing for routine surveillance (regardless of clinical signs)
- ◯ Testing based on the presence of clinical signs (e.g., cough, sneeze, diarrhea, mortality)
- ◯ Testing based on other factors. Please explain:

____

13. When you collect oral fluids for routine surveillance, how many ropes do you hang per barn/pigs? Please tell us number of ropes per barn and number of pigs.

____

14. When you collect oral fluids based on presence of clinical signs (e.g., cough, sneeze, diarrhea, mortality etc), how many ropes do you hang per barn/pigs? Please tell us number of ropes per bar and number of pigs.

____

15. What best describes the method you use to determine the number of ropes to hang?

- ○ Number dictated by company/clinic guidelines
- ○ I decide how many ropes I need to hang
- ○ Other. Please explain: ____

16. What is the average number of ropes used per pen?

- ○ One per pen
- ○ One per 2 pens (hung on the gating separating 2 pens)
- ○ Two per pen
- ○ Other. Please explain: ____

17. What is the average number of pens sampled per barn?

____

18. What would be the average number of pigs represented by a single oral fluid-rope sample?

____

Sampling and Sample Management Procedures

19. Who usually collects the oral fluid sample?

- ○ I do
- ○ Caretaker for the site
- ○ Company/clinic health technician
- ○ Regional production supervisor
- ○ Other. Please explain: ____

20. Roughly, what is the length of time pigs have access to the rope? Enter total of minutes below:

____

21. Do you know what is the source (the store, vendor the rope originated from) and the diameter of the rope?

- ☐ Source: ____
- ☐ Diameter (please include units): ______________________________

22. Do you have a written protocol for handling/processing the ropes to collect oral fluid samples at the farm?

- ◯ YES
- ◯ YES and farm personnel has a copy
- ◯ YES but farm personnel does not have a copy
- ◯ NO

23. Once samples are collected, are they immediately:

- ◯ Stored in refrigeration at the farm
- ◯ Stored in refrigeration in veterinarian/regional production supervisor truck
- ◯ Left in veterinarian/regional production supervisor truck under refrigeration until arrive at the office immediately after visit
- ◯ Left in veterinarian/regional production supervisor truck under refrigeration until arrive at office at the end of the day after other farm visits
- ◯ Other. Please explain: ____

24. How much time (average, minimum, maximum) goes by between oral fluid collection and submission to the diagnostic laboratory?

- ☐ Average (Hours) ____
- ☐ Minimum (Hours) ____
- ☐ Maximum (Hours) ____
- ☐ I don't know

Sample Submission

25. Once samples are collected, where do they go next?

- ○ My sample processing lab at company or clinic and then shipped to VDL
- ○ Directly to the VDL from farm
- ○ Other. Please explain: ______________________________

26. When oral fluid samples are submitted, which of the following information do you include? (Check all that apply)

- ☐ Clinical signs observed
- ☐ Number of dead pigs
- ☐ Total number of pigs in the site
- ☐ Pig age
- ☐ Number of pigs with clinical signs
- ☐ The date on which clinical signs were first observed
- ☐ Pen(s) and barn (s) where oral fluids originated from
- ☐ The Premises Identification Number **(PIN)**
